# Tobacco cessation among smokers under substance use treatment for alcohol and/or cannabis: study protocol and pilot study

**DOI:** 10.1186/s13722-022-00348-9

**Published:** 2022-11-30

**Authors:** Ariadna Feliu, Esteve Fernández, Yolanda Castellano, Marta Enríquez, Judith Saura, Carmen Cabezas, Joan Colom, Josep M. Suelves, Margarida Pla, Mar Parejo, Sílvia Mondon, Pablo Barrio, Magalí Andreu, Antonia Raich, Jordi Bernabeu, Jordi Vilaplana, Xavier Roca, Pablo Bautista, Joseph Guydish, Cristina Martínez, group of clinicians involved in the recruitment of the study, Laia Miquel, Pol Bruguera, Karen Nadal, Monika Anduaga, Silvia Martínez, Beth Pallejà, Rosanna Reyes, Enrique Surribas, Francina Fonseca, Clara Caterina, Diego Aranega, Nuria Cabezón, Víctor Martí, Amalia Gual, Carolina Franco, Delia Parellada, Laura Masferrer, Esther Batllori

**Affiliations:** 1 Tobacco Control Unit, Cancer Control and Prevention Program, WHO Collaborating Center On Tobacco Control, Institut Català d’Oncologia, L’Hospitalet de Llobregat, Barcelona, Spain; 2 Cancer Control and Prevention Group, Institut d’Investigació Biomèdica de Bellvitge-IDIBELL, L’Hospitalet de Llobregat, Barcelona, Spain; 3 CIBER en Enfermedades Respiratorias, CIBERES), Madrid, Spain; 4 Department of Clinical Sciences. School of Medicine and Health Sciences, University of Barcelona, Barcelona, Spain; 5 Department of Public Health, Maternal Health and Mental Health, School of Medicine and Health Sciences, Universitat de Barcelona, L’Hospitalet del Llobregat, Barcelona, Spain; 6 Government of Catalonia, Public Health Secretariat, Barcelona, Spain; 7 Public Health Agency of Catalonia, Barcelona, Spain; 8 Universitat Oberta de Catalunya, Barcelona, Spain; 9 Addictions Unit, Psychiatry Department, Institute of Neurosciences, Hospital Clínic de Barcelona, Barcelona, Spain; 10 Mental Health Department, Althaia Xarxa Assistencial Universitària, Manresa, Barcelona, Spain; 11 Serra Húnter Fellow, Computer Science Department, Universitat de Lleida, Lleida, Spain; 12 Addictive Behaviors Unit, Psychiatry Department, Hospital de La Santa Creu I Sant Pau, Barcelona, Spain; 13 Philip R. Lee Institute for Health Policy Studies, University of California San Francisco, San Francisco, CA 94158 USA

**Keywords:** Cannabis, Alcohol, Smoking cessation, Substance abuse

## Abstract

**Background:**

Approximately 80% of people with a substance use disorder (SUD) are smokers. Starting SUD treatment offers the opportunity to also quit smoking. The ACT-ATAC project aims to identify the predictors associated with smoking cessation among persons treated for alcohol and/or cannabis use disorder in Barcelona. This manuscript reports its methodology and the experience of carrying it out during the COVID-19 pandemic.

**Methods:**

Mixed methods project with three substudies. Substudy 1 (S1) comprises heterogeneous discussion groups among clinicians. S2 has two prospective cohorts composed of smokers under treatment for alcohol and/or cannabis use disorder and the clinicians in charge of these patients. Participating smokers will be followed for 12 months and interviewed about their substance use and the tobacco cessation services received using the Spanish version of the users’ Knowledge, Attitudes, and Services (S-KAS) scale. The clinicians will be asked about their self-reported practices in smoking cessation using the Knowledge, Attitudes, and Practices (S-KAP) scale. S3 comprises heterogeneous discussion groups with smokers. Data will be triangulated using qualitative and quantitative analyses. To facilitate the recruitment process, the researchers have introduced several strategies (design clear protocols, set monthly online meetings, extend the project, provide gift cards, etc.).

**Discussion:**

The results of S1 were used to develop the questionnaires. S2 required some adjustments due to the COVID-19 pandemic, particularly the follow-up interviews being conducted by phone instead of face-to-face, and the recruitment rhythm was lower than expected. Recruitment will last until reaching at least 200–250 users. The fieldwork could not have been possible without the collaboration of the ACT-ATAC team and the introduction of several strategies.

***Trial registration*** The ACT-ATAC project has been successfully registered at Clinicaltrials.gov [NCT04841655].

## Background

Tobacco use is the single most preventable morbidity and mortality [1]. Though progress has been made in reducing the prevalence of tobacco consumption in the general population [2], high prevalence (75.2% to 85.1%) remains in some vulnerable populations, particularly among people with substance use disorders (SUDs) [3]. Tobacco is responsible for 53% of the deaths in this group, reducing their life expectancy up to 25 years compared to the general population [4].

Smokers with SUDs take up smoking at an early age, consume more cigarettes per day (CPD), and frequently use different tobacco products [3], resulting in increased nicotine dependence [5] and a two to five times lower likelihood of cessation [6]. In SUD treatment settings, tobacco use has been historically considered a minor problem compared to other substances. Smoking was even once considered therapeutic in preventing substance use relapse [7]. People in treatment for SUDs have shown interest in quitting smoking [8] and make more serious attempts (abstinence  ≥ 24 h) when they are offered both psychological and pharmacological assistance [9].

Approximately 81,000 people annually are admitted to specialized treatment centers in Spain for outpatient treatment for SUDs [10]. Alcohol is responsible for four out of 10 admissions, and cannabis for two out of 10. Data from the Spanish Drug Observatory indicate that 44.7% of people admitted to outpatient treatment for alcohol and 83.3% admitted for cannabis use disorder have used tobacco in the previous 30 days [11]. However, only 44% of the SUD Programs in Catalonia (Spain) provide tobacco cessation treatment as part of their portfolios [12].

Given the high concomitant use of tobacco in patients in SUD treatment, its consequences to physical and mental health, and the low deployment of interventions in Spain, there is an urgent need to study which factors promote the introduction of smoking cessation in these programs.

Therapeutic strategies for smoking cessation come from recent studies conducted mainly in the United States and Australia. These studies suggest a variety of therapeutic targets, including the promotion of smoking reduction as an initial goal [13, 14], the combination of cognitive-behavioral interventions and existing pharmacotherapy [15], relapse prevention [16]), extended follow-ups [6], and a comprehensive model in SUD treatment settings and the community that responds to the concomitant use of tobacco and other substances [13].

The optimal time to promote smoking cessation in people with SUDs is controversial [17, 18], mainly due to patients' low motivation to quit and their concerns about how quitting smoking will affect their treatment for quitting the main drug(s) [19]. Thus, some clinicians are reluctant to provide interventions that address tobacco use during SUD treatment [20]. Recent experiences, however, suggest that concurrent interventions are well accepted by smokers being treated for both alcohol [21] and cannabis use [22, 23], reporting satisfactory 7-day smoking abstinence rates similar to the general population (28%) [24] and a lower relapse rate than among those who continue smoking [25].

Simultaneously quitting both substances has both psychological and neurobiological benefits [14]. This approach may also be beneficial from an economic point of view as both treatments can be targeted during a single treatment episode, reducing costs. Yet, simultaneous interventions are less frequent than consecutive interventions. A previous study conducted in Barcelona (Spain) found that smokers with alcohol use disorder who were offered smoking cessation achieved better quit rates for both alcohol and tobacco when both drugs were treated simultaneously [26].

Exploration of the barriers and facilitators to abstinence and understanding what elements affect the psychiatric symptomatology and the main drug use are relevant to patients in SUD treatment. Acquiring such information will allow the scientific community to design personalized approaches to promote cessation [27, 28] within this vulnerable group. Previous studies associated smoking cessation in this population with sociodemographic variables (i.e., low education level, low socioeconomic status) [29] and other components, such as motivation to quit smoking, self-efficacy, previous experience with quitting smoking [30], expected withdrawal symptoms [31], and assessment of the services received [9]. Proactively identifying positive predictors of change among smokers in this population may help promote smoking cessation; the most frequently reported predictors are a high level of motivation, having had previous quit attempts, and reducing the number of CPD [13, 14].

Barriers to addressing tobacco use during addiction treatment have also been identified among clinicians. The main barriers are lack of knowledge and training, the preconception that quitting smoking simultaneously while being treated for use of other drugs may compromise abstinence, and clinicians’ smoking status [20].

The high number of SUD treatment admissions for alcohol and/or cannabis use, the high prevalence of tobacco use, and the limited number of interventions aimed at promoting smoking cessation in this group prompts research to facilitate new and better interventions in these settings. We designed the ACT-ATAC (“*Abandono del Consumo de Tabaco durante la Atención y el Tratamiento para Alcohol y/o Cánnabis*” [Tobacco cessation among smokers under alcohol and/or cannabis treatment]) study, which aims to:

(1) Explore clinicians' perceptions of the barriers and facilitators (individual and contextual) that influence the implementation of smoking cessation interventions, and the appropriateness of simultaneous or sequential interventions aimed at this group.

(2) Describe changes in tobacco use, motivation to quit, and the barriers/facilitators to quit at the start of treatment and after 12 months of follow-up.

(3) Identify the individual and contextual predictors associated with successful smoking cessation among persons treated for alcohol and/or cannabis use disorder in SUD programs in Barcelona.

(4) Identify and compare the level of implementation of smoking cessation services reported by patients and clinicians.

The purpose of this manuscript is to report the methodology employed in the ongoing ACT-ATAC project and the experience of carrying out and adjusting this study during the social restrictions put in place due to the COVID-19 pandemic.

## Methods

### Design

**Fig. 1:**
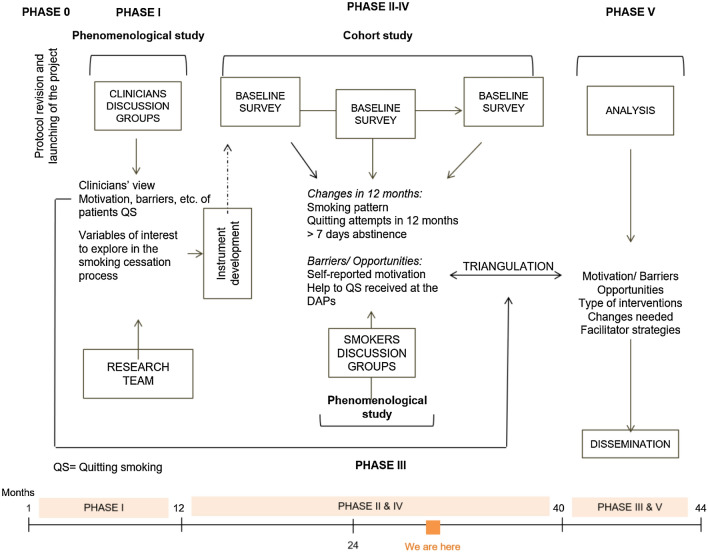
The ACT-ATAC project flowchart

We designed a mixed method observational study (Fig. 1). The ACT-ATAC project began in January 2019 and was conceived as a 36-month study. However, due to the COVID-19 pandemic, we have extended the study for eight more months. At the present time, December 2021, we are in month 35 (Fig. 1) and we foresee the study lasting until the end of June 2023.

Mixed method studies include both a qualitative and a quantitative research perspective in which one of the study designs may have a lead role and the other has a more supporting role [32]. In this project, the cohort study is the primary design. The study is divided into the following substudies (S1-S3).

S1 (months 1–11): Phenomenological study with discussion groups directed towards health professionals (corresponding to January 2019–November 2019; carried out before the outbreak of COVID-19 in Spain).

This qualitative phenomenological study with discussion groups used a semi-structured script to answer aim 1. Our target was all healthcare professionals working at one of the 42 SUD programs in the province of Barcelona. Twelve centers were chosen from those that expressed interest according to a geographical distribution criterion to ensure variability of areas, as 65% of the centers in the province belong to the city of Barcelona and the rest to other nearby cities. Clinicians from these SUD centers were recruited via a letter signed by the Head of the Catalan Drug Plan and addressed to program directors, asking them to engage their staff in participating.

### Participants

Participants included all healthcare professionals from the selected SUD programs who work in the mental health field with at least 1 year of experience in the field and agreed to participate and provide informed consent. Clinicians were grouped into three heterogeneous discussion groups (8 to 10 participants each) led by two experts in qualitative research who followed a script of themes to be explored. Themes included (1) barriers and opportunities to address tobacco use among persons in treatment for alcohol and/or cannabis use; (2) health professionals’ opinions on the type of smoking cessation interventions that could be offered to these patients and whether these interventions should be simultaneous or consecutive with the treatment of the main drug, and (3) experiences in helping smokers quit.

### Procedures and statistical analysis

Sessions were recorded and transcribed, preserving participant anonymity and confidentiality, to conduct a thematic categorical content analysis (AC-CT). Thematic axes were proposed to finish constructing the questionnaire needed in the second phase of the study.

S2 (months 11–40): Longitudinal prospective study with smokers in treatment for alcohol and/or cannabis and the clinicians involved (corresponding to November 2019–December 2022; study piloted before the outbreak of COVID-19 in Spain but carried out during and after the severe lockdown of March 2020-June 2020, and currently ongoing).

The second substudy covers aims 2 and 3 and consists of two prospective cohorts composed of (1) smokers in treatment for alcohol and/or cannabis who visited one of the participating SUD programs, who agreed to participate in this study, and had at least one responsible healthcare professional to recruit participants during the first and second year of the study, and (2) the clinicians in charge of these patients.

### Cohort of smokers

#### Participants

Inclusion criteria are (1) being a tobacco user (at least one cigarette per week), (2) initiating treatment for alcohol and/or cannabis in one of the SUD participating program, (3) age  ≥ 18 years, (4) under follow-up by participating clinicians, and (5) providing consent. Exclusion criteria are (1) inability to guarantee a 12-month follow-up (due to anticipated mobility problems, etc.) and (2) severe cognitive impairment and/or other communication problems (i.e., not speaking Spanish or Catalan).

#### Sampling method

The incidence of unassisted smoking cessation is seven ex-smokers per 100 patient-years [33]. We estimate that, among this sample, being in contact with the health system may increase this rate to 10 ex-smokers per 100 patient-years (i.e., a relative risk [RR] of quitting smoking of 2.0). Therefore, to detect this RR assuming an alpha error of 5%, a beta error  < 20%, and losses of 20%, we require 366 participants. The sample size was calculated using the GRANMO program [34]. This sample size is achievable in one year as the 48 centers in the province of Barcelona treat approximately 5000 new cases for alcohol and 1500 new cases for cannabis yearly (target population: 6,500 participants) [12]. On average, the 10–12 selected study centers should treat approximately 1,625 people/year. Smokers in treatment for alcohol and/or cannabis use are being recruited consecutively for one year.

#### Recruitment process and fieldwork

**Fig. 2:**
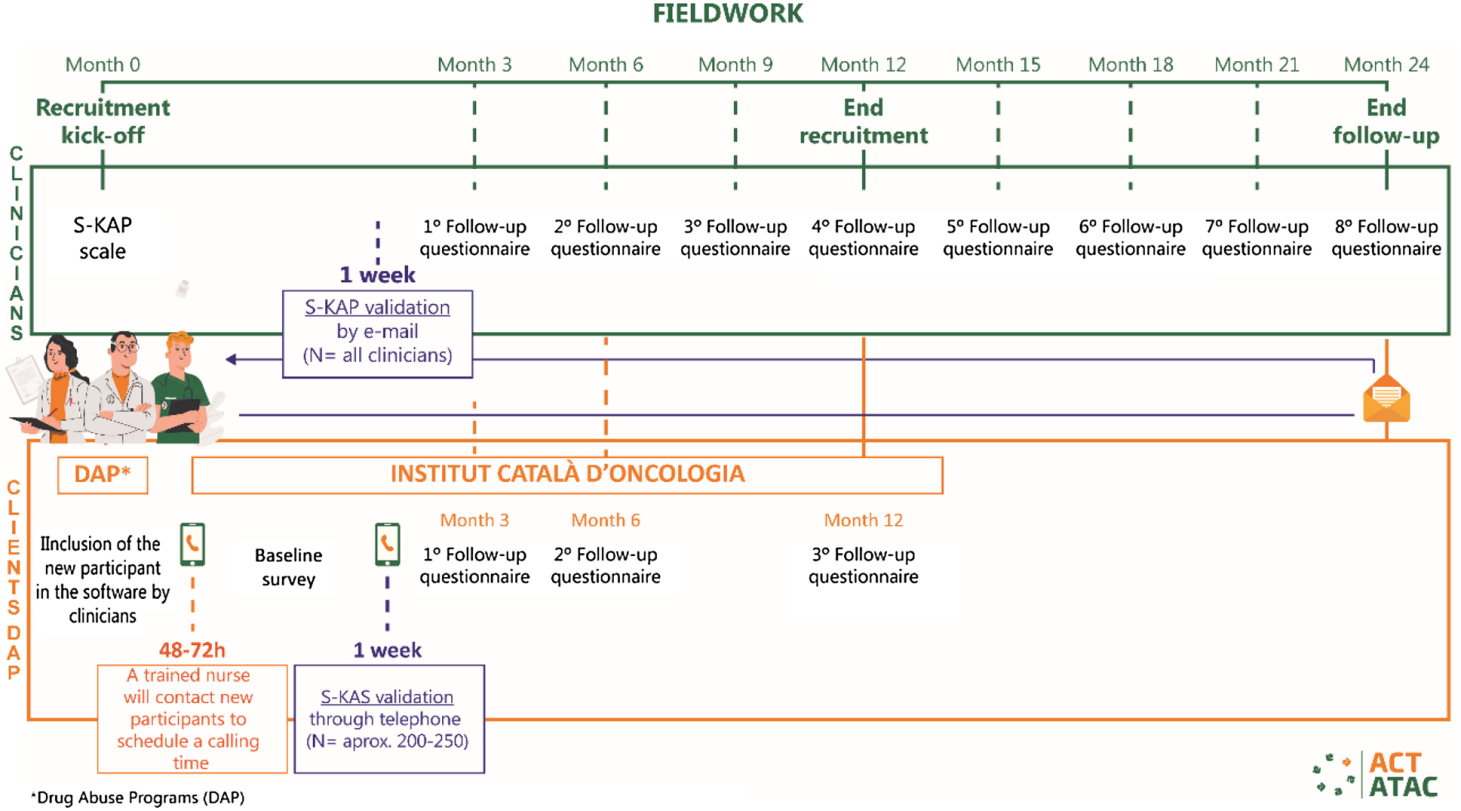
Fieldwork diagram

Clinicians working in the participating centers determine whether their patients meet the inclusion criteria described above and invite those patients to be part of the study. Those who accept to participate and complete informed consent procedures are registered using computer software designed for the ACT-ATAC project, including personal identifiers (i.e., name, surname), psychiatric diagnoses, comorbidities, and location data (contact telephone numbers, responsible clinician, clinician's telephone number, etc.). To facilitate the recruitment process, a protocol has been created in the software. After the participant’s data is introduced into the software, he/she is contacted in the first 48–72 h by a research nurse who conducts an extensive interview (see below). The study nurse is independent to any SUD participating center and was recruited exclusively to conduct this interviews. The study procedures are aimed at following up on the tobacco consumption of patients and other variables. The research nurse also informs the patient when he/she will be contacted for follow-up interviews by telephone (at 7 days and 3, 6, and 12 months; Fig. 2).

#### Fieldwork and strategies implemented to overcome the barriers due to the COVID-19 pandemic

**Fig. 3:**
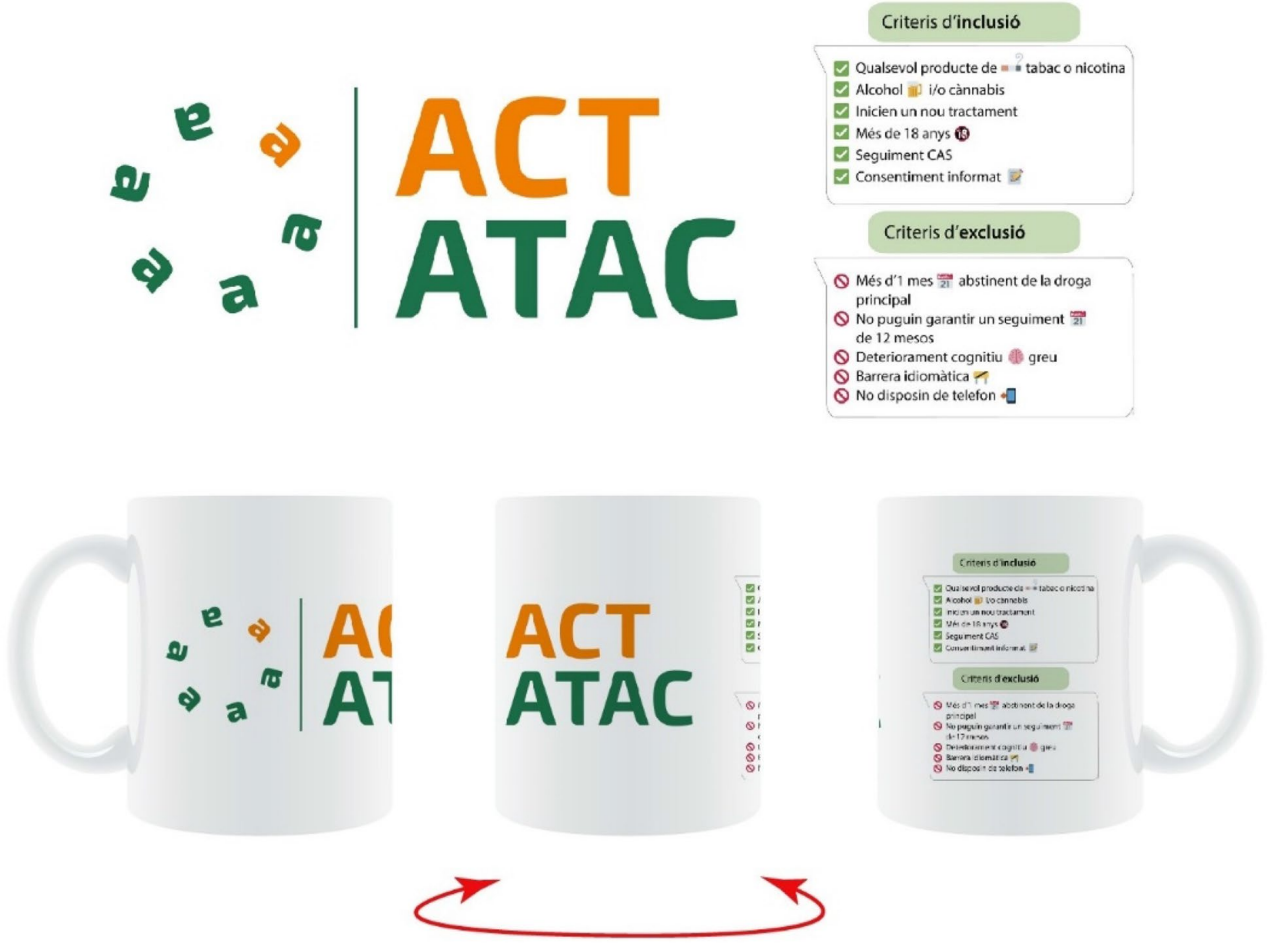
Clinician’s mug with the inclusion and exclusion criteria

The longitudinal fieldwork kicked-off in November 2019 (month 11). The first step of the fieldwork was recruiting SUD centers and working clinicians willing to participate in the project. The pilot study was conducted between November 2019 and February 2020 and allowed us to test the recruitment process and software designed to collect and store data, as well as ensure the correct follow-up of participants. Nevertheless, in March 2020, the COVID-19 pandemic in Spain forced us to modify the follow-up interviews from face-to-face to telephone-based to limit the social contact of the research nurse with the participants. In addition, the rhythm of patient inclusion in the study slowed down from 1 or 2 per week before the lockdown (from March to June 2020) to 2 or 3 per month. Despite this, the study was able to be continue because the SUD centers in Catalonia remained open, though many of the visits were conducted online. Thus, the collaborating clinicians were still able to recruit patients who met the inclusion criteria during the pandemic; however, we recommended that they did so during in-person meetings to better assess participants’ cognitive status and make getting their informed consent easier. These adjustments were possible thanks to the collaboration between the study coordinators, the collaborating clinicians, and the Direction of the Substance Abuse Program at the Public Health Agency in Catalonia, that prioritized our project and contacted clinicians to keep this study on track despite the contextual circumstances. To increase the engagement of clinicians (clinical psychologists, psychiatrists, and nurses), we have conducted monthly online meetings with them to exchange experiences and difficulties in the recruitment process and given them a certificate of participation in the study signed by the Director of the Substance Abuse Program at the Public Health Agency in Catalonia and the Principal Investigator. These meetings are an informal 30-min coffee break in which investigators and clinicians get together and share their experiences regarding the recruitment process and present the latest information on the participants’ profile. A mug with the main inclusion and exclusion criteria has been designed and gifted to clinicians for them to pick up during these meetings (Fig. 3).

Moreover, to improve participants’ fidelity in the follow-up study, halfway through the study, we started offering 20 euros gift cards to those who answer all of the phone calls during the 12-month follow-up. At the time of submitting this manuscript, 98 patients have been recruited. Thus, we will continue the study until we recruit at least 200 to 250 participants (expected by the end of 2022).

#### Variables

Dependent variables include tobacco use, cannabis use, and alcohol use. Tobacco use is rated yes/no. If answering “yes”, we ask the about the tobacco consumption pattern, including nicotine dependence using the 6-item Fagerstrom test [35], and the type of tobacco or other products consumed (i.e., cigarettes, roll-your-own tobacco, combination of both, cigars, e-cigarettes, iQOS); smoking history, including the age when the participant starting smoking, maximum abstinence time, previous quit attempts, and resources used (i.e., none, self-help, professional support, pharmacological treatment, etc.); motivation to quit smoking, measured by a Likert scale from 0 to 10; and willingness to quit smoking, assessed by a 4-item questionnaire that, according to Prochaska and DiClemente’s model, classifies smokers into five stages: pre-contemplative, contemplative, preparation, action, and maintenance [36].

Cannabis use is rated yes/no. If “yes”, we recorded users pattern of consumption, problematic use (yes/no) [37], withdrawal symptoms identified using the Marijuana Craving Questionnaire [38] and reasons for use [39]. Alcohol use is rated yes/no and described according to the variables of consumption included in the Alcohol Use Disorders Identification Test (AUDIT) [40, 41]. A fourth dependent variable is participants’ self-reported smoking knowledge, attitude, and services (S-KAS) received during their treatment in SUD programs [42].

The primary outcome is abstinence from smoking (yes/no), which is self-reported and verified by the exhaled CO concentration only if they report to be abstinent. Self-reported abstinence is defined as “no smoking in the 7 days prior to the assessment point.” Seven-day abstinence was chosen as the consumption measure based on the consensus recommendation in practice guidelines for the assessment of abstinence in patients who are not motivated to quit smoking and who may remain abstinent during various periods of the study [43]. Number of quit attempts in the last year of  > 24-h duration [44] were also recorded. All dependent variables are assessed at baseline and 3, 6, and 12 months of follow-up (Table 1).

**Table 1:** Study variables and collection time points (at baseline and follow-up)

Variables	Follow-up							
	Baseline	1 week	3 months		6 months		12 months	
			Abstinent	Not abstinent	Abstinent	Not abstinent	Abstinent	Not abstinent
**Tobacco**								
Type of tobacco or other products consumed and quantity	X			X		X		X
Nicotine dependence	X			X		X		X
Age at first consumption	X							
Age at start of regular consumption	X							
Previous attempts to stop smoking in their entire consumption trajectory	X							
Previous attempts to quit smoking in the last year	X							
Previous attempts to quit smoking since the last questionnaire				X		X		X
Maximum withdrawal time	X							
Resources used to quit smoking	X		X	X	X	X	X	X
Motivation to quit smoking	X			X		X		X
Smoking cessation provision	X		X	X	X	X	X	X
Efficacy	X		X	X	X	X	X	X
Level of smoking approach received	X	X	X	X	X	X	X	X
Environment	X							
Abstinence from consumption	X		X	X	X	X	X	X
**Cannabis**								
Consumption once in lifetime	X		X		X		X	
Consumption in the last month	X		X	X	X	X	X	X
Frequency of cannabis use in the last month	X			X		X		X
Problem cannabis use (only for patients under treatment for alcohol abuse)	X		X	X	X	X	X	X
Frequency of use, consumption, and interaction with tobacco in the last 30 days	X		X	X	X	X	X	X
Context of cannabis smoking	X			X		X		X
Latest consumption	X		X	X	X	X	X	X
Age at start of consumption	X							
Reasons for cannabis use	X							
Previous attempts to stop using cannabis	X							
Maximum withdrawal time	X							
Resources used to stop using cannabis	X							
Willingness to quit cannabis use	X		X	X	X	X	X	X
Motivation to quit cannabis use	X			X		X		X
Efficacy	X		X	X	X	X	X	X
Environment	X							
**Alcohol**								
Alcohol consumption in the last month	X		X	X	X	X	X	X
Problem alcohol use (only patients receiving cannabis dishabituation)	X							
Context of alcohol drinking	X							
Latest consumption	X		X	X	X	X	X	X
Age at start of consumption	X							
Reasons for alcohol consumption	X							
Previous attempts to stop drinking alcohol	X							
Maximum withdrawal time	X							
Resources used to stop drinking alcohol	X							
Willingness to quit alcohol	X		X	X	X	X	X	X
Motivation to quit alcohol	X			X		X		X
Efficacy	X		X	X	X	X	X	X
Environment (family, friends, etc.)	X							
**Psychiatric diagnostic**								
Suicide risk	X				X	X		
Quality of life	X				X	X	X	X
Self-perceived health status	X		X	X	X	X	X	X
Sociodemographic	X							
**Primary outcome**								
Breathed CO concentration	X		X	X	X	X	X	X

Independent variables include sociodemographic data (sex, age, employment status and occupation, and education level), self-perceived state of health addressed by the question “How would you say your general health is?”, measures of mental and physical health according to the DSM-V [45], quality of life according to the General Health Questionnaire (GHQ-12) [46], and level of previous smoking cessation treatment received using questions about standard interventions included in the Smoking Cessation Guidelines (i.e., advice, reduction recommendation, educational material, cognitive-behavioral treatment, pharmacological treatment, nothing) [47].

#### Instrument

Baseline and follow-up questionnaires have been designed including all variables and their dimensions described above. These questionnaires are based on the findings of the discussion groups with clinicians and an extensive literature research. The backwards translation of the S-KAS [42] and the Marijuana Withdrawal Questionnaire from English to Spanish was performed by two Spanish native speakers. All data are entered into a computer software designed for the ACT-ATAC project for the introduction and management of patient data. The software allows recruitment and recording of initial variables by the clinician, and introduction of the results of the questionnaire interviews carried out by the field nurse. The initial and follow-up questionnaires (6 and 12 months) are not accessible by the clinician.

### Cohort of clinicians

#### Participants

Clinicians from the province of Barcelona who agree to participate in the study must meet the following inclusion criteria: (1) to be a clinician (psychologist, psychiatric, nurse, social worker, etc.) in one of the public SUD programs in the province of Barcelona, and (2) that does the initial assessment and follow-up of persons with alcohol and/or cannabis abuse disorders, and (3) provides consent. The exclusion criterion is an inability to guarantee a 12-month follow-up (due to anticipated moves, etc.). We expected to include between 20 and 30 clinicians, and 22 clinicians are currently collaborating in the study from 10 different SUD centers.

#### Variables

The primary dependent variable is self-reported smoking knowledge, attitude, and practice (S-KAP) [48] during the clinician’s work in the SUD program, monitored at baseline of their participation, one week after, and every three months during their participation in the study.

Independent variables are tobacco consumption (never user, former user, and current user), sociodemographic data (sex, age, education level, profession), and professional experience (years treating drug abuse population, years working in the same program). In the case of being a tobacco user, we ask about the tobacco product consumed (i.e., cigarettes, roll-your-own tobacco, combination of both, cigars, e-cigarettes, iQOS); smoking history, including age when starting smoking, maximum abstinence time, previous quitting attempts, and resources used (i.e., none, self-help, professional support, pharmacological treatment, etc.); motivation to quit smoking, measured by a Likert scale from 0 to 10; and willingness to quit smoking, assessed by a 4-item questionnaire according to Prochaska and DiClemente’s model, classifying smokers into five stages of change [36].

Clinicians receive the KAP questionnaire [48] every three months to report data on the approach taken in the care of the smoker (ask, advise, assist).

## Statistical analysis

After finishing the recruitment and follow-up, abstinence and quit attempt incidence rates will be calculated at baseline and at 3, 6, and 12 months according to whether participants have received any type of smoking cessation intervention. We will also calculate the corresponding risk ratios with 95% confidence intervals (CIs) for quitting and quit attempts.

We will assess the association between receiving smoking cessation support and the two primary outcomes (abstinence and quit attempts) using a Poisson regression model with robust variance for several independent variables (the substance of treatment, sex, types of cessation support [e.g., tobacco-related counselling and medication, etc.]), resulting in RRs with their 95% CIs as their estimators. For analysis, we will use SPSS for Windows version 21 with significance set at p  < 0.05.

We will also conduct exploratory factor analysis for testing the psychometric properties of the S-KAS and S-KAP scales.

S3 (months 40–44): Phenomenological study with discussion groups directed towards patients (will be conducted between December 2022 and June 2023).

This qualitative phenomenological study with discussion groups will use a semi-structured script to answer aim 4.

### Participants

Participants for the qualitative study will be sampled from the cohort of smokers in S2 according to intensive sampling for theoretical representativeness and maximum variation. The selection criteria will be the dimensions from the 6-month follow-up questionnaire. The number of participants will be oriented towards meeting two requirements: that each testimonial has the potential to help understand the area/topic studied, and that the number of testimonials registered saturates the information collected. This threshold is met when participant themes are repeated. The selection criteria have been chosen to determine different profiles based on sex, age, main consumption, and smoking profile (previous quit attempts and receipt of help to quit smoking in the SUD program). Groups will be arranged according to four different dimensions of homogenization.

### Data collection and variables

We will conduct eight homogeneous discussion groups with 60 to 90-min duration. The sessions will be recorded and transcribed to preserve participants’ anonymity and confidentiality. During these sessions, two experts on qualitative research will explore the barriers and opportunities to quit smoking, experiences with the approach to smoking that participants received during their treatment for alcohol and/or cannabis use, and their opinion on the service received.

### Analysis

We will conduct a thematic categorical content analysis (TC-CA) applying the following rigor criteria suggested by Guba and Lincoln [49]: credibility, dependence, and confirmability.

### Ethical considerations

The research protocol has been submitted to and approved by the Clinical Research Ethics Committee (CREC) of the University Hospital of Bellvitge [PR315/20] and the CREC of each participating organization. Participants will receive a verbal explanation of the aims of the study and a written study information sheet, and will be asked to provide written informed consent. The ACT-ATAC project follows the standards of Law 3/2018 on the protection of personal data. The data collected in the computer application will be secure and will require personal passwords. The ACT-ATAC project is also registered at Clinicaltrials.gov [NCT04841655].

## Discussion

Over the course of the study, the COVID-19 pandemic forced us to adjust some parts of the study without changing its purpose or the core elements of its methodology. The most notable adjustment has been to replace face-to-face follow-up interviews with telephone-based interviews and to extend the study another year to ensure we recruit enough participants to have enough statistical power to conduct the analysis. All of the changes have been possible thanks to the successful collaboration between the coordinators, the clinicians, and the Public Agency of Public Health in Catalonia. The ACT-ATAC team has worked together in setting up multiple videocalls to keep the study on track.

In addition to the pandemic, another significant challenge faced by the ACT-ATAC research group was designing ad hoc baseline and follow-up questionnaires, particularly regarding the description of type of products and pattern of consumption for cannabis. Unlike the measurement for alcohol use, there are few validated questionnaires designed to monitor the consumption of cannabis (alone and in combination with tobacco). Therefore, we conducted a thorough review following recent recommendations [50–52] and developed a battery of questions to address the mode and frequency of consumption (i.e., marijuana, hashish, CBD oil) and its interaction with tobacco products in the past 30 days. The validity of these questions will be tested in the study and will be shared with the scientific community.

Moreover, we have included two scales used in the US to assess “Smoking-related Knowledge, Attitudes, and Services (KAS)” among users [42] and “Smoking-related Knowledge, Attitudes, and Practices (KAP)” [48] among providers. These scales were validated in the US, but their validity has not been assessed in Spanish. Therefore, we decided to translate them and validate their psychometric proprieties for the Spanish population. This work will allow us to have a valid Spanish language instrument to measure relevant variables in the tobacco control dimensions related to the views of users and staff. This validation is an added value for mental health professionals and decision-makers, as these scales could be of help in understanding how smoking cessation services are provided in SUD programs.

Furthermore, by describing individual and contextual predictors of tobacco use among alcohol and/or cannabis users treated for SUDs, we will better understand the factors that influence individuals to continue or quit smoking and the primary drug of use (alcohol and/or cannabis). Understanding which of these factors are associated with higher smoking and alcohol/cannabis cessation rates is key to informing the Spanish healthcare system’s future intervention programs based on the theory of change [53]. This would allow healthcare planners and stakeholders to design better evidence-based programs that consider the characteristics of the users and clinicians involved, helping to reduce the burden of smoking in this vulnerable population.

## Limitations and strengths

A key study limitation may be the limits on extrapolating the findings, as it is complex to understand the use of tobacco and other drugs in a way that represents all SUD clinicians and patients, and we do not know the extent to which the pandemic has affected the attendance of SUD programs. However, the mixed methods design includes several heterogeneous discussion groups for both clinicians and patients with different broad typologies in order to capture a broad range of experiences, barriers, and opportunities and how the pandemic has impacted them. Regarding the questionnaires, self-reported data may include reporting or cognitive biases from participants. Moreover, on account of the patient-professional relationship, selection bias is possible among recruiting clinicians, who could offer the study only to patients who they believe will agree to participate. Lastly, conducting the follow-up interviews by telephone instead of face-to-face may affect the quality of the data collected. However, we had no other option due to the COVID-19 pandemic.

Despite its limitations, the study includes a mixed methods exploratory approach, represents both patient and clinician perspectives, permits triangulation of multiple data sources, and measures primary outcomes (abstinence and quit attempts) in a 1-year follow-up design.

Finally, we strongly believe that the adjustments that were made have allowed us to carry out the study despite the pandemic. Complex observational studies allow the study of real-life phenomena; however, researchers may need to overcome difficulties, such as the COVID-19 pandemic, by introducing innovative strategies to successfully carry out these studies [54]. We are confident that studying tobacco use among the SUD population under these circumstances will allow us to design programs that can be sustained in the real life of clinicians and patients.

## Data Availability

Not applicable.
